# MUC1 Selectively Targets Human Pancreatic Cancer in Orthotopic Nude Mouse Models

**DOI:** 10.1371/journal.pone.0122100

**Published:** 2015-03-27

**Authors:** Jeong Youp Park, Yukihiko Hiroshima, Jin Young Lee, Ali A. Maawy, Robert M. Hoffman, Michael Bouvet

**Affiliations:** 1 Department of Surgery, University of California San Diego, San Diego, California, United States of America; 2 AntiCancer, Inc., San Diego, California, United States of America; 3 Department of Internal Medicine, Yonsei University College of Medicine, Seoul, Korea; 4 Department of Surgery, Yokohama City University Graduate School of Medicine, Yokohama City, Japan; 5 Surgical Service, VA San Diego Healthcare System, San Diego, California, United States of America; National Cancer Institute, UNITED STATES

## Abstract

The goal of this study was to determine whether MUC1 antibody conjugated with a fluorophore could be used to visualize pancreatic cancer. Anti-MUC1 (CT2) antibody was conjugated with 550 nm or 650 nm fluorophores. Nude mouse were used to make subcutaneous and orthotopic models of pancreatic cancer. Western blot and flow cytometric analysis confirmed the expression of MUC1 in human pancreatic cancer cell lines including BxPC-3 and Panc-1. Immunocytochemistry with fluorophore conjugated anti-MUC1 antibody demonstrated fluorescent areas on the membrane of Panc-1 cancer cells. After injecting the conjugated anti-MUC1 antibodies via the tail vein, subcutaneously transplanted Panc-1 and BxPC-3 tumors emitted strong fluorescent signals. In the subcutaneous tumor models, the fluorescent signal from the conjugated anti-MUC1 antibody was noted around the margin of the tumor and space between the cells. The conjugated anti-MUC1 antibody bound the tumor in orthotopically-transplanted Panc-1 and BxPC-3 models enabling the tumors to be imaged. This study showed that fluorophore conjugated anti-MUC1 antibodies could visualize pancreatic tumors in vitro and in vivo and may help to improve the diagnosis and treatment of pancreatic cancer.

## Introduction

The tumor marker CA19-9 can be detected in the serum of patients with pancreatic cancer. However, the usefulness of CA19-9 as a diagnostic or prognostic cancer biomarker is questionable. The sensitivity of serum CA19-9 ranges from 41 to 86% with a specificity of 33 to 100%, which is not suitable for screening or diagnosis [1]. CA19-9 is frequently elevated in other inflammatory diseases and bile obstruction conditions such that its usefulness as a biomarker is even more questionable. Better biomarkers for pancreatic cancer are needed [2].

MUC1 is a membrane-bound glycoprotein consisting of a large extracellular subunit of a 20 amino acid tandem repeat domain, a small extracellular domain subunit, a transmembrane domain and a cytoplasm tail [3]. MUC1 is frequently overexpressed in various cancers including breast, ovarian, lung, and colon cancer [3,4]. It is also considered as a potential diagnostic, prognostic, and therapeutic biomarker of pancreatic cancer. MUC1 is overexpressed in over 90% of pancreatic cancer patient tumors [5]. Strong expression of MUC1 is associated with reduced survival [6]. MUC1 targeted therapy has been tested in preclinical and clinical trials [7–9]. Attempts have been made to detect MUC1 in the serum of patients and pancreatic cancer tissue with various methods [10,11].

We have demonstrated that anti-CEA antibody conjugated with fluorophores helped to improve cancer detection and enabled fluorescence-guided surgery (FGS) in pancreatic and colon cancer mouse models which significantly improved outcome compared to standard bright-light surgery [12–14]. It has been reported that cathepsin and claduin-4 targeted optical imaging helped to detect pancreatic cancer and its precursor in mouse models [15,16].

In the present study, we determined whether anti-MUC1 antibody conjugated with a fluorophore could target and visualize pancreatic cancer in vitro and in vivo models.

## Materials and Methods

### Pancreatic cancer cell lines

The human pancreatic cancer cell lines BxPC-3 [17] and Panc-1 [18] were maintained in RPMI 1640 medium supplemented with 10% fetal bovine serum (Hyclone, Logan, UT), penicillin/streptomycin (Gibco-BRL, Carlsbad, CA), sodium pyruvate (Gibco-BRL), sodium bicarbonate (Cellgro, Manassas, VA), L-glutamine (Gibco-BRL), and minimal essential medium nonessential amino acids (Gibco-BRL). All cells were cultured at 37° C in a 5% CO_2_ incubator.

### Construction of GFP-expressing pancreatic cancer cell line

The construction of green fluorescent protein (GFP) expressing Panc-1 cell line was done as described previously [19]. For GFP gene transduction, 20% confluent Panc1 cells [18] were incubated with a 1:1 precipitated mixture of retroviral supernatants of the PT67 packaging cells and RPMI 1640 (Gibco-BRL, Life Technologies, Inc.) for 72 h. The cells were harvested by trypsin/EDTA 72 h after incubation with GFP retroviral supernatants and subcultured at a ratio of 1:15 into selective medium that contained 200 μg/ml G418. The level of G418 was increased to 800 μg/ml stepwise. Clones expressing GFP were isolated with cloning cylinders (Bel-Art Products, Pequannock, NJ) by trypsin/EDTA and were amplified and transferred by conventional culture methods. High GFP-expression clones were then isolated in the absence of G418 for > 10 passages to select for stable expression of GFP [20–22].

### Mice

Athymic *nu/nu* nude mice (AntiCancer Inc., San Diego, CA), 4–6 weeks old, were used in this study. Mice were kept in a barrier facility under HEPA filtration. Mice were fed with an autoclaved laboratory rodent diet. All mouse surgical procedures and imaging were performed with the animals anesthetized by intramuscular injection of 50% ketamine, 38% xylazine, and 12% acepromazine maleate (0.02 ml). Animals received buprenorphine (0.10 mg/kg ip) immediately prior to surgery and once a day over the next 3 days to ameliorate pain. The maximum tumor size was less than 2 cm. The condition of the animals was monitored every day. The animals were all sacrificed 2–3 weeks after surgery. CO2 inhalation was used for euthanasia. To ensure death following CO2 asphyxiation, cervical dislocation was performed. All animal studies were approved by AntiCancer, Inc.’s Institutional Animal Care and Use Committee (IACUC) in accordance with the principals and procedures outlined in the National Institute of Health Guide for the Care and Use of Animals under Assurance Number A3873-1.

### Antibody-dye conjugation

Hamster monoclonal antibodies to MUC1 (CT2; Thermo Scientific, Rockford, IL, USA) were conjugated with DyLight 650 or 550 dyes (Thermo Scientific, Rockford, IL, USA) per manufacturer specifications, ensuring a minimum of at least 4:1 dye: protein ratio. Protein: dye concentrations were confirmed using a NanoDrop Spectrophotometer (Thermo Fisher Scientific, Waltham, Massachusetts) [23].

### Western blotting

Cell lysates were extracted in lysis buffer containing 70 mM β-glycerophosphate, 0.6 mM sodium orthovanadate, 2 mM MgCl_2_, 1 mM ethylene glycol tetraacetic acid, 1 mM DTT (Invitrogen, Grand Island, NY, USA), 0.5% Triton-X100, 0.2 mM phenylmethylsulfonyl fluoride, and 1% protease inhibitor cocktail (Sigma-Aldrich, St. Louis, MO, USA). Lysates were separated by sodium dodecylsulfate–polyacrylamide gel electrophoresis (SDS-PAGE) and transferred to polyvinylidene fluoride membranes (Millipore, Billerica, MA, USA). The membranes were blocked in 5% (w/v) non-fat dry milk and probed with antibodies. Anti-MUC1 (CT2) was used. The immunoreactive proteins were visualized using the SuperSignal West Pico Chemiluminescent Substrate (Thermo Scientific, Rockford, IL, USA).

### Flow cytometric analysis

Cells were grown to 80% confluence, treated with accutase solution (Sigma) for 5 minutes, washed twice with fluorescence-activated cell sorting (FACS) buffer (2% FBS, 0.1% sodium azide in PBS). Cells (1 × 10^6^) were resuspended in the FACS buffer. Cells were fixed with 2% paraformaldehyde and then blocked with 2% BSA-PBS solution for 30 min at room temperature. Cells were incubated with anti-MUC1 (CT2, 2 μg/test) for 40 min at room temperature, and then with goat anti-Armenian hamster IgG-FITC (Santa Cruz Biotechnology, Dallas, TX, USA) for 40 min at room temperature. After washing with FACS buffer, cells were resuspended in FACS buffer and subjected to flow cytometry using a FACS Aria (BD Immunocytometry Systems, Franklin Lakes, NJ). Side scatter and forward scatter profiles were used to eliminate cell doublets.

### Immunocytochemistry of live cells

Panc-1 cells (2 x 10^5^) were cultured overnight. Anti-MUC1 (CT2) antibody conjugated with DyLight 550 dyes was diluted to 4 μg/ml in PBS (Corning cellgro, Manassas, VA). The culture medium from the cells was aspirated and the diluted antibody was added to the live cells. Cells were incubated with antibody for 1 hour at room temperature. The cells were washed gently 2 times with phosphate-buffered saline after the antibody was aspirated. The cells were observed under an FV1000 confocal microscope (Olympus, Tokyo, Japan) with white light and 559 nm laser [24].

### Immunohistochemistry

For fluorescence immunostaining on slides made from frozen tumor tissue, anti-MUC1 (CT2) conjugated with DyLight 650 was used. The slides were incubated with 10% normal donkey serum for 1 hour at room temperature, and incubated with the conjugated antibody at room temperature for 1 hour at a dilution of 1:100. Tissues were dried and observed with an IV-100 scanning laser microscope (Olympus, Tokyo, Japan) with 633 nm laser [25]. Alternate slides from the same frozen tumor tissue were stained with hematoxylin and eosin and observed under light microscope.

### Skin flap model

Nude mice were anesthetized with the ketamine mixture via subcutaneous injection. An arc-shaped incision was made in the abdominal skin. The subcutaneous connective tissue was separated to free the skin flap without injuring the artery and vein. The skin flap was spread and fixed on the flat stand. Panc-1-GFP (1 X 10^6^ cells) in 30 μl of matrigel were sprinkled on the inside surface of the skin flap, and the skin was closed [26].

### Subcutaneous and orthotopic tumor mouse model

To make subcutaneously transplanted pancreatic tumor models, Panc-1 and BxPC-3 human pancreatic cancer cells (2 × 10^6^) were injected subcutaneously into the flanks of nude mice. When the subcutaneous tumors grew between 10 and 20 mm in diameter, they were harvested and fragmented into small fragments. The tumor fragments were implanted orthotopically in nude mice, as previously described [19,27–29].

### Whole body imaging

In both subcutaneous and orthotopic tumor models, the mice were injected with the antibody conjugated with fluorophore into the tail vein, and then whole body imaging was performed using the OV100 Small Animal Imaging System (Olympus, Tokyo, Japan) after anesthesia with the ketamine mixture described above [30]. The optimal dose for animal studies was decided by dose of the conjugated antibody which produced images with the best tumor to background ratio in subcutaneous tumor model.

### Image analysis

All images were analyzed using Image-J (National Institutes of Health, Bethesda, Maryland) before the process of images and compared. Image process was done with Adobe Photoshop CS3 (Adobe Systems Inc., San Jose, California).

## Results

### Expression of MUC1 in pancreatic cancer cell lines

**Fig 1:**
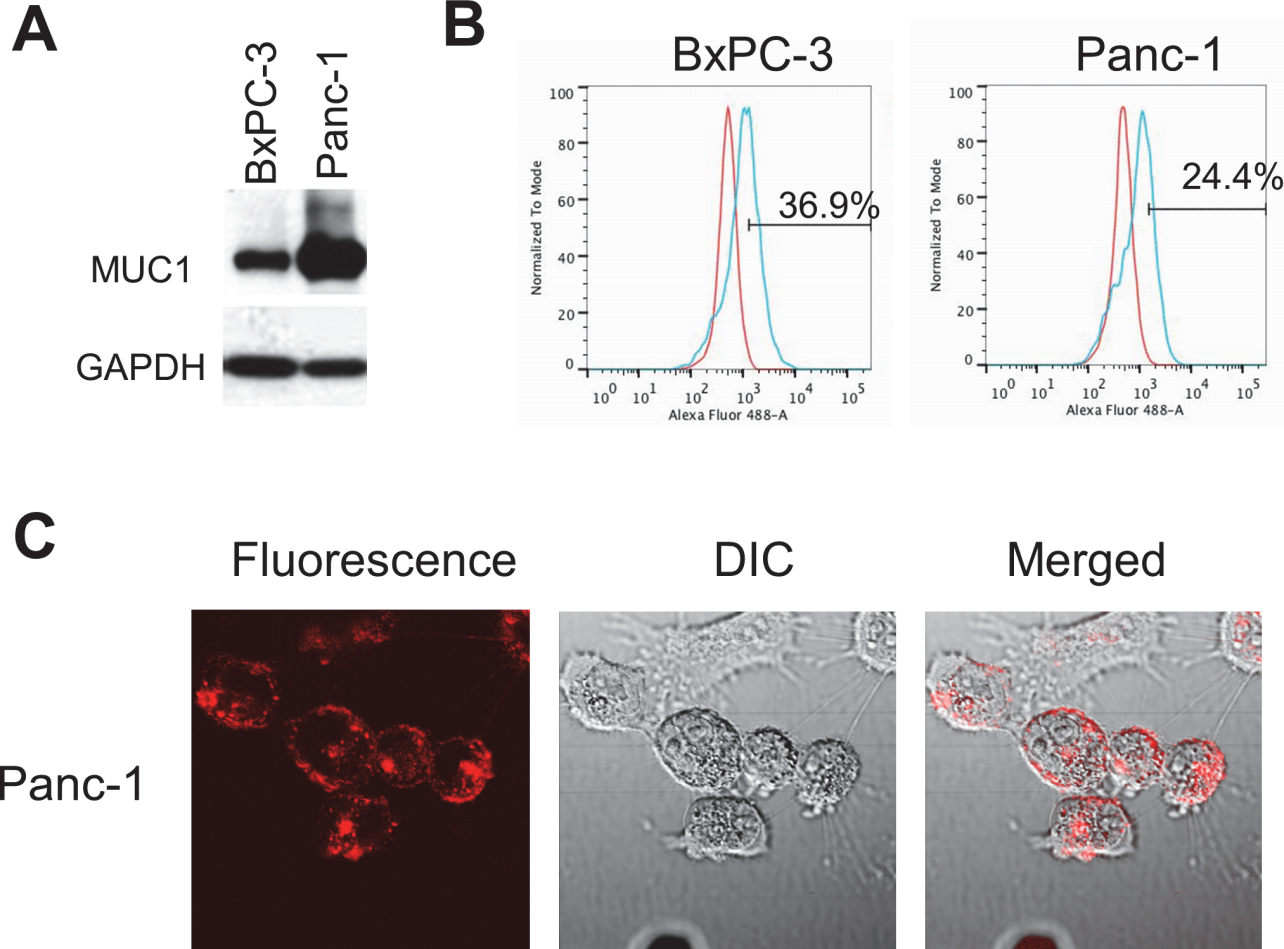
Characterization of pancreatic cancer cell lines. (A) Western blot analysis shows MUC1 expression in pancreatic cancer cell lines (BxPC-3 and Panc-1). (B) Flow cytometric analysis shows the expression of MUC1 on the surface of BxPC-3 and Panc-1 cell lines. (C) Immunocytochemistry on live cells shows multiple fluorescent dots on the surface of Panc-1 cells. Representative fluorescence images merged with corresponding DIC (differential interference contrast) images (x60 water immersion objective on FV1000, using the 559 nm laser).

Both pancreatic cancer cell lines tested (BxPC-3 and Panc-1) had MUC1 expression observed with Western blotting (Fig 1A). The proportion of MUC1 positive cells were 36.9% in BxPC3 and 24.4% in Panc1 pancreatic cancer cell lines, as observed with flow cytometry (Fig 1B). The same test was repeated in each cell line at least three times. The results of all experiments showed that anti-MUC1 (CT2) antibody could detect MUC1 on the cell membrane of pancreatic cancer cells in vitro.

Immunocytochemistry was also performed using anti-MUC1 (CT2) antibody conjugated with flourophores. After incubation with the conjugated antibody without permeation, multiple fluorescent dots were noted on the surface of Panc-1 cells under a fluorescence microscope (Fig 1C). Merging with corresponding differential interference contrast microscopy confirmed that the fluophore-conjugated antibody reacted with MUC1 on the surface of the pancreatic cancer cells and produced fluorescence.

### Targeting of subcutaneous pancreatic tumors with fluorescent MUC1

**Fig 2:**
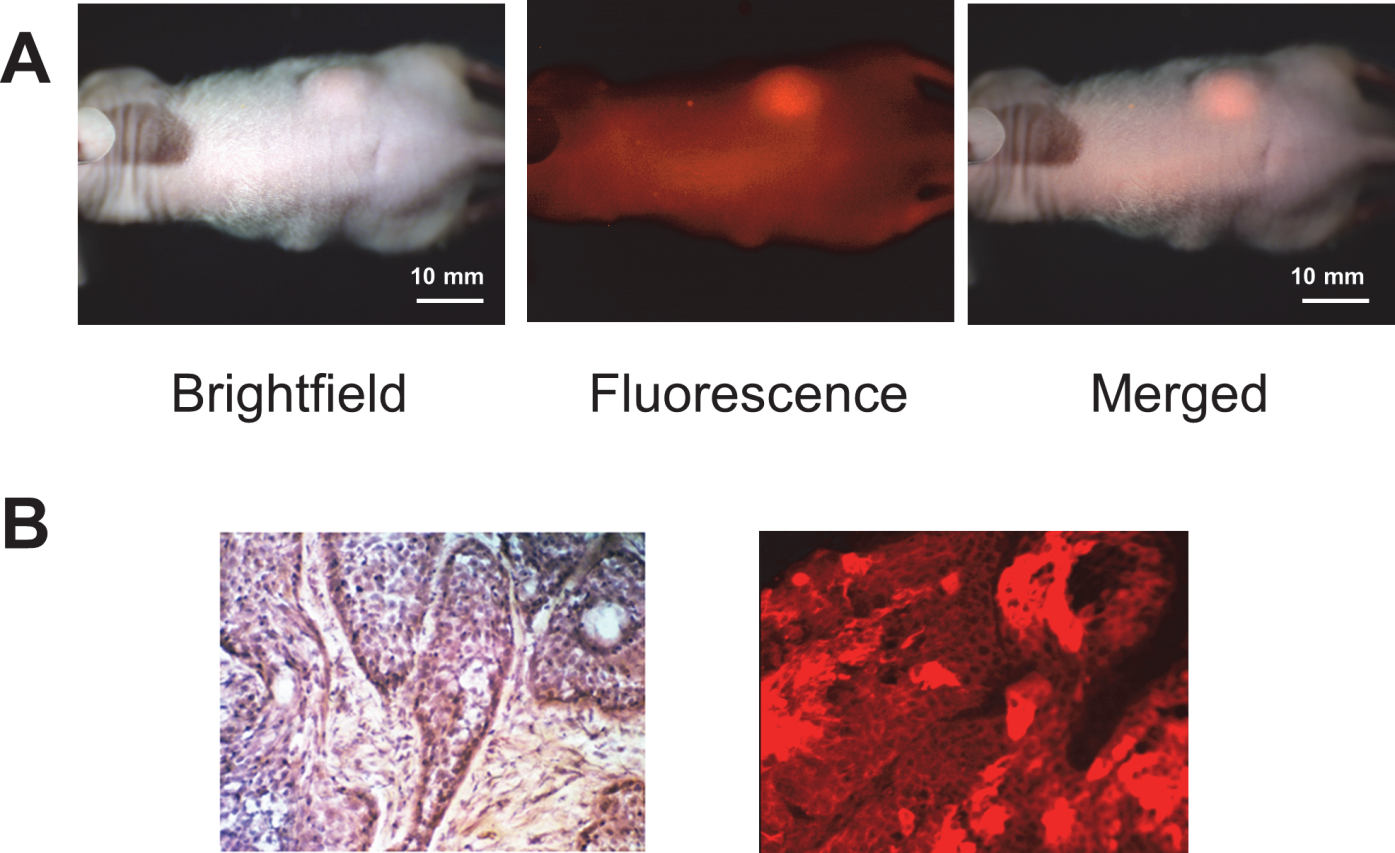
Imaging of MUC1 targeting of subcutaneously-transplanted pancreatic tumors in vivo. Representative images are shown. (A) The mouse is imaged under both white light and fluorescence illumination. The intensity of the red fluorescence signal from the Panc1 subcutaneous tumor is stronger than background. (B) Hematoxylin and eosin staining (x200, left). Fluorescence immunostaining for MUC1 (x20 regular objective, right) of frozen tumor samples shows fluorescence signals at the membrane of the cancer cells.

When BxPC-3 or Panc-1 subcutaneous tumors had reached approximately 10–20 mm in diameter, the animals were each given a single 30 μg dose of DyLight 650-conjugated anti-MUC1 (CT2) via the tail vein. The mice were imaged under both brightfield and fluorescence illumination using the Olympus OV100 Small Animal Imaging System. Both Panc-1 (Fig 2A) and BxPC-3 (data not shown) tumor showed stronger fluorescence. Fluorescence immunostaining was performed on frozen tumor samples made from BxPC-3 and showed fluorescence on cell membranes demonstrating the expression of MUC1 (Fig 2B).

### Targeting of pancreatic cancer cells in skin flaps with fluorescent MUC1

**Fig 3:**
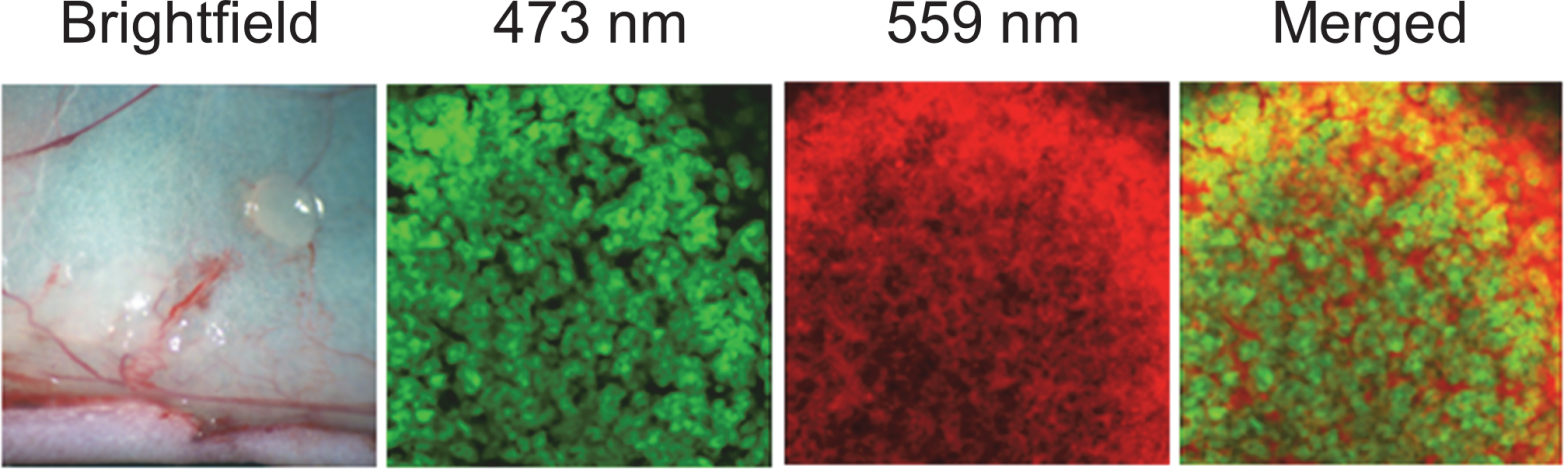
Imaging of MUC1 targeting of pancreatic tumors growing on skin flaps. Representative images were obtained under white light, and 473 nm and 559 nm lasers on the OV100, and merged. GFP signal from the individual cancer cells and red fluorescent signal from the DyLight 550 fluorophore-conjugated anti-MUC1 antibody at the outside margin of the colony and space between the cancer cells were observed.

Mice with pancreatic cancer cells growing in skin flaps were injected with anti-MUC1 (CT2) conjugated with DyLight 550 dyes into the tail vein. Forty-eight hours after the antibody injection, the skin flap was spread again. Imaging was performed with the OV100 and FV1000. Several colonies of cancer cells were noted on the surface of skin flap with the OV100. GFP fluorescence signals from individual cancer cells were observed with the FV1000. At the outside margin of the colony and space between the cancer cells inside of the colony, 550 nm fluorescence signals, which originated from the fluorophore-conjugated antibody, were observed. (Fig 3) The results demonstrated that the antibody reacted with MUC1 on the membrane of the cancer cells in vivo.

### Targeting of pancreatic tumors in orthotopic models with fluorescent MUC1

**Fig 4:**
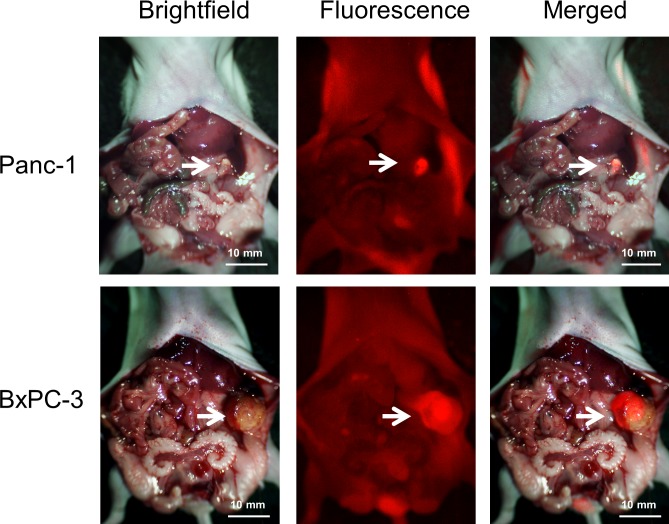
Imaging of MUC1 targeting of orthotopically-transplanted Panc-1 and BxPC-3 pancreatic tumor in vivo. Fluorescence signals from pancreatic tumors orthotopically transplanted at the tail of the pancreas were detected. Other than the tumor, fluorescence signal was detected from the skin and, bladder and intestinal contents but at lower intensity than the tumor. White arrows indicate pancreatic tumor.

Mice with tumors implanted orthotopically into the mouse pancreas were injected with DyLight 650-conjugated anti-MUC1 (CT2) antibody with a single 30 μg dose via the tail vein 7–10 days after tumor implantation. Imaging was performed after opening the abdomen of the mice. Intravital imaging with the OV100 detected the fluorescence signal coming from Panc1 and BxPC3 tumors (Fig 4). Tumors less than 5 mm could be detected with fluorescence imaging (Fig 4). The mean ratios of fluorescence intensity between tumor and background in Panc-1 and BxPC-3 orthotopic tumors were 6.70 and 2.39, respectively. These results confirmed that in orthotopic tumor models, MUC1 targeted pancreatic cancer and enabled tumor detection. Other than the tumor, fluorescence signals were detected from the skin and, bladder and intestinal contents, but at lower intensity.

## Discussion

MUC1 is a very attractive imaging biomarker for pancreatic cancer since it is overexpressed in approximately 90% of pancreatic cancer patients [5,6]. The present results from subcutaneous and orthotopic tumor models demonstrate that MUC1 can target and visualize pancreatic cancer by making it fluorescent. The results of the present study demonstrate that MUC1 is an additional target for pancreatic cancer labeling and therapeutics delivery. MUC1 may be a useful target to identify and treat distant metastasis of all cancer types expressing the antigen using labeled or therapeutic-carrying antibodies.

BxPC-3 and Panc-1 were selected based on previous studies with these cell lines for fluorescence-guided surgery (FGS) [13,14]. It is possible that the fraction of positive cells in the tumor is less than that seen by flow cytometry due to steric hindrance to antibody entry into the tumor. It is also possible that the presence of stromal cells in the tumor may decrease the percentage of positive cells.

The CT2 antibody is recognizing both live cancer cells as well as the cells within the tumor in the mouse which is readily visualized by conjugation of the antibody to a fluorophore. It is possible that in the live cell, sufficient parts of the cytoplasmic tail become accessible to the antibody. As recently shown by Kumar et al, more study is needed to understand structure and subcellular localization of MUC1 protein [31].

There have been attempts to use MUC1 as a treatment target. MUC1 targeted imaging can guide therapeutics to the tumor [11,32,33]. Imaging for MUC1 can demonstrate whether therapeutic targets are present in cancer cells and predict the treatment response of MUC1-targeted therapy [5]. In addition, fluorescence imaging targeted at MUC1 in pancreatic cancer can improve tumor visualization in order to achieve complete resection during surgery. Fluorescent guided surgery (FGS) has been shown to improve survival in mouse models with pancreatic cancer [13,14].

In the present study, we wished to study MUC1 targeting of human pancreatic cancer with the next goal of studying MUC1 targeting in our pancreatic cancer orthotopic xenograft (PDOX) models [12]. Subsequent future studies will be on MUC1 targeting in spontaneous PDAC mouse models. Formal bio-distribution studies will be done in future experiments. The present study shows MUC1 can be used for selective tumor targeting in vivo. Based on the ability of fluorescent MUC1 antibody targeting to illuminate these tumors, we can now compare FGS in future studies with labeled anti-CEA and anti-MUC1.
